# Real-world effectiveness of medications on survival in patients with COPD-heart failure overlap

**DOI:** 10.18632/aging.102004

**Published:** 2019-06-07

**Authors:** Vincent Yi-Fong Su, Yao-Hsu Yang, Diahn-Warng Perng, Ying-Huang Tsai, Kun-Ta Chou, Kang-Cheng Su, Wei-Juin Su, Pau-Chung Chen, Kuang-Yao Yang

**Affiliations:** 1 Department of Internal Medicine, Taipei City Hospital, Taipei City Government, Taipei, Taiwan; 2 Department of Chest Medicine, Taipei Veterans General Hospital, Taipei, Taiwan; 3 Faculty of Medicine, School of Medicine, National Yang-Ming University, Taipei, Taiwan; 4 Institute of Clinical Medicine, National Yang-Ming University, Taipei, Taiwan; 5 Institute of Emergency and Critical Care Medicine, National Yang-Ming University, Taipei, Taiwan; 6 Cancer Progression Research Center, National Yang-Ming University, Taipei, Taiwan; 7 Division of Pulmonary and Critical Care Medicine and Department of Respiratory Care, Chang Gung Memorial Hospital, Chiayi, Taiwan; 8 Health Information and Epidemiology Laboratory, Chang Gung Memorial Hospital, Chiayi, Taiwan; 9 Department of Traditional Chinese Medicine, Chang Gung Memorial Hospital, Chiayi, Taiwan; 10 School of Traditional Chinese Medicine, College of Medicine, Chang Gung University, Taoyuan, Taiwan; 11 Department of Respiratory Therapy, Chang Gung University, Taoyuan, Taiwan; 12 Institute of Occupational Medicine and Industrial Hygiene, National Taiwan University College of Public Health, Taipei, Taiwan; 13 Department of Environmental and Occupational Medicine, National Taiwan University Hospital and National Taiwan University College of Medicine, Taipei, Taiwan

**Keywords:** chronic obstructive pulmonary disease, heart failure, COPD-heart failure overlap, long-acting β2 agonist, long-acting muscarinic antagonist

## Abstract

The appropriate treatment for patients with coexistent chronic obstructive pulmonary disease (COPD) and heart failure (HF) remains unclear. Data from the Taiwan National Health Insurance Research Database was used for this retrospective cohort study. Patients diagnosed with both diseases between 1997 and 2012 were enrolled as the COPD-heart failure overlap cohort. Patients were categorized as non-users and users of specific COPD and HF medications. Medication prescriptions in each 3-month and 1-year period served as time-dependent covariates. The primary endpoint was cumulative survival. The validation study confirmed the accuracy of definitions of COPD (94.0% sensitivity) and HF (96.3% sensitivity).

The study included 275,436 patients with COPD-heart failure overlap, with a mean follow-up period of 9.32 years. The COPD-heart failure overlap cohort had more medical service use and higher mortality than did the COPD alone cohort. Use of inhaled corticosteroid (ICS)/long-acting β2 agonist (LABA) combinations, long-acting muscarinic antagonist (LAMA), angiotensin receptor blockers (ARBs), β blockers, aldosterone antagonists, and statins reduced mortality risk compared with non-use. Sensitivity and subgroup analyses confirmed the consistency and robustness of results.

ICS/LABA combinations, LAMA, ARBs, β blockers, aldosterone antagonists, and statins use was associated with a lower mortality risk in patients with COPD-heart failure overlap.

## INTRODUCTION

Chronic obstructive pulmonary disease (COPD) and heart failure (HF) are major global health issues characterized by high mortality and morbidity [1, 2]. The COPD-heart failure overlap leads to important therapeutic challenges. The cornerstones of COPD therapy are long-acting inhaled bronchodilators (anticholinergics and β2 agonists) with or without inhaled corticosteroids (ICSs) [1]. Improvements in medical treatment have shifted the treatment of HF from the use of positive inotropic agents to efforts to limit and slow its progression [2]. Angiotensin-converting enzyme inhibitors (ACEIs), angiotensin receptor blockers (ARBs), β blockers, and aldosterone antagonists reduce hospitalization and mortality in patients with HF.

Although β blockers are established therapy for HF, this class of drugs is often underused in patients with COPD-HF overlap [3]. Furthermore, patients receiving ARBs were less likely to have cough compared with those receiving ACEIs [4]. ACEI-related cough could lead to bronchial hyperresponsiveness [5]. Moreover, the presence of bronchial hyperresponsiveness in COPD is associated with increased mortality [6]. More evidence of the survival benefit of this therapy is needed. Therefore, the aim of this study was to investigate the survival effects of medications recommended in guidelines for patients with COPD-heart failure overlap using a population-based nationwide dataset.

## RESULTS

### Clinical characteristics of the study population

**Figure 1:**
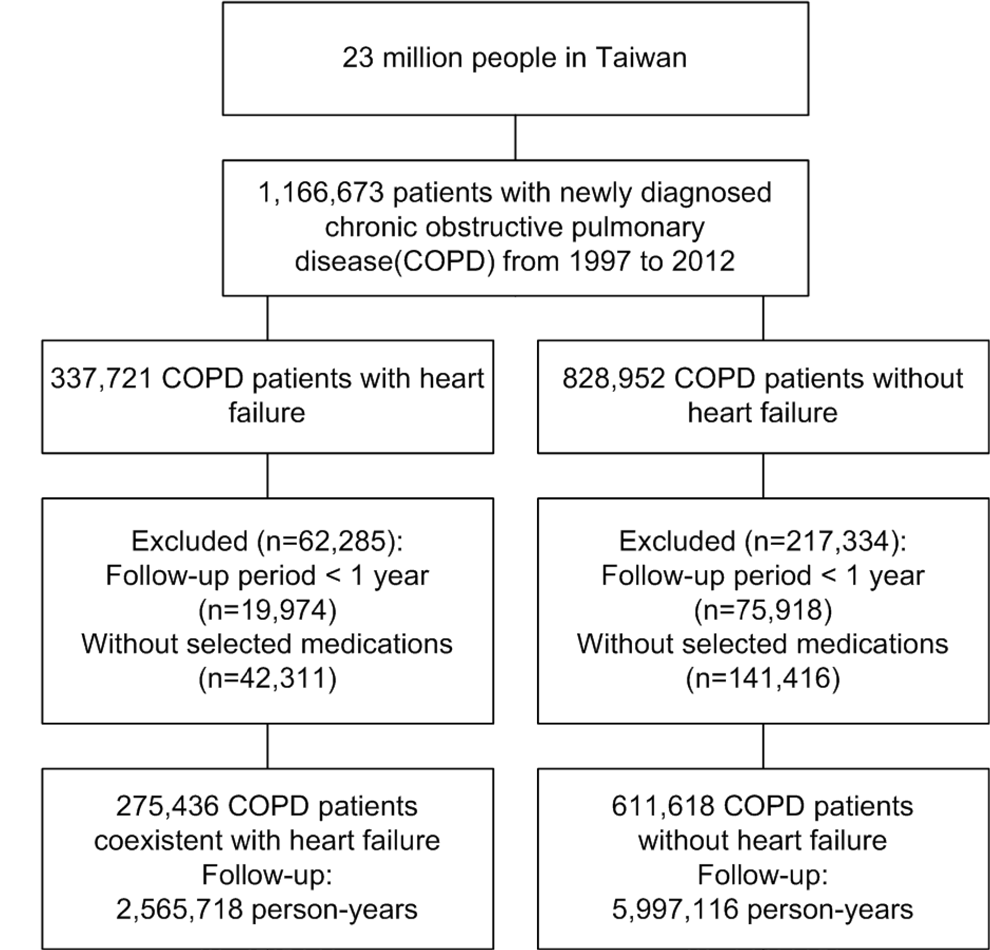
**Flow diagram summarizing the process of enrollment.**

**Figure 2:**
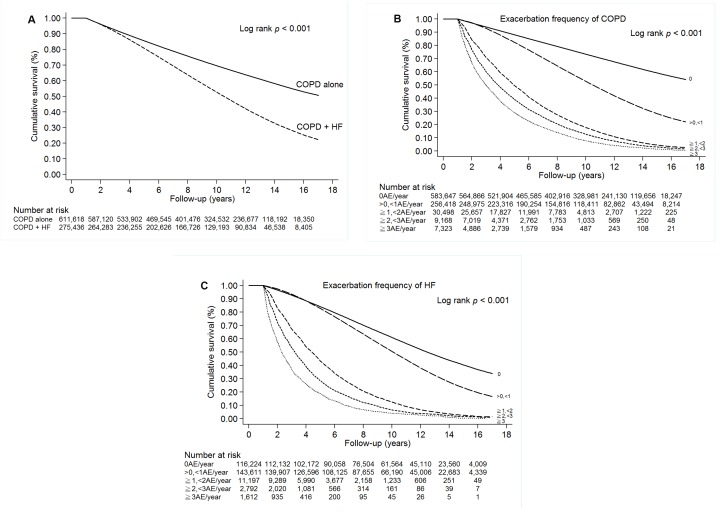
(**A**) Percentage of patients with COPD-heart failure overlap (COPD+HF) in different age groups. (**B**) Percentage of patients with COPD-heart failure overlap (COPD+HF) in different age groups in male sex. (**C**) Percentage of patients with COPD-heart failure overlap (COPD+HF) in different age groups in female sex.

After the application of exclusion criteria, the study cohort consisted of 611,618 patients with COPD alone and 275,436 patients with COPD-heart failure overlap (Figure 1). The mean follow-up periods for the COPD-heart failure overlap and COPD alone cohorts were 9.32 and 9.81 years, respectively. Table 1 shows the baseline characteristics of the cohorts. The percentage of COPD-heart failure overlap patients increased significantly with age at the time of COPD diagnosis; the greatest proportion of these patients was aged ≥80 years (total, 45.90%; male, 40.56%; female, 53.66%; Figure 2A–2C). Compared with the COPD alone cohort, the COPD-heart failure overlap cohort was older (mean age, 64.38 *vs.* 70.63 years; *p* < 0.0001) and contained fewer men (64.02% *vs.* 59.14%; *p* < 0.0001). Compared with the COPD alone cohort, the COPD-heart failure overlap cohort had more outpatient visits (mean, 2.44 *vs.* 2.79), hospitalizations for COPD or HF (mean, 0.15 *vs.* 0.46; all *p* < 0.0001) per year.

**Table 1:** Characteristics of the COPD-heart failure overlap cohort and COPD alone cohort.

**Characteristics**	**COPD-heart failure overlap cohort**		**COPD alone cohort**		***p* value**
	***n***	**%**	***n***	**%**	
N	275436		611618		
Age, years (mean ± SD)	70.63±9.87		64.38±11.83		<.0001
Age			<.0001
<65	65199	23.67	287901	47.07	
≥65	210237	76.33	323717	52.93	
Follow-up, years(mean ± SD)	9.32±4.26		9.81±4.26		<.0001
Sex			<.0001
Female	112546	40.86	220045	35.98	
Male	162890	59.14	391573	64.02	
Comorbidities					
Diabetes mellitus	101903	37.00	180785	29.56	<.0001
Hypertension	224020	81.33	359818	58.83	<.0001
Cerebrovascular disease	76127	27.64	121677	19.89	<.0001
Ischemia heart disease	143048	51.94	189921	31.05	<.0001
Malignancy	39000	14.16	105500	17.25	<.0001
Cirrhosis	68138	24.74	171537	28.05	<.0001
Chronic kidney disease	14457	5.25	8962	1.47	<.0001
Charlson Comorbidity Index					
0–1	87422	31.74	294082	48.08	<.0001
>1	188014	68.26	317536	51.92	<.0001
Urbanization					
I	58921	21.39	157047	25.68	<.0001
II	113363	41.16	267800	43.78	<.0001
III	64095	23.27	115765	18.93	<.0001
IV	39057	14.18	71006	11.61	<.0001
Income level					
0	64631	23.47	111657	18.26	<.0001
1–15840	61442	22.31	117733	19.25	<.0001
15841–25000	129684	47.08	286852	46.90	<.0001
≧25000	19679	7.14	95376	15.59	<.0001
OPD visit/year(first year)	2.79±4.96		2.44±4.26		<.0001
Exacerbation frequency of COPD (AE/year)					
0 AE/year	134269	48.75	449378	73.47	<.0001
>0, <1 AE/year	117834	42.78	138584	22.66	<.0001
≧1, <2 AE/year	15114	5.49	15384	2.52	<.0001
≧2, <3 AE/year	4586	1.66	4582	0.75	<.0001
≧ 3AE/year	3633	1.32	3690	0.60	<.0001
Exacerbation frequency of HF (AE/year)					
0 AE/year	116224	42.20			
>0, <1 AE/year	143611	52.14			
≧1, <2 AE/year	11197	4.07			
≧2, <3 AE/year	2792	1.01			
≧ 3AE/year	1612	0.58			
Hospitalization for COPD and HF/year (mean ± SD)	0.46±0.75		0.15±0.49		<.0001
COPD Medications					
SABDs	271527	98.58	592993	96.95	<.0001
LABAs alone	69496	25.23	201216	32.90	<.0001
ICSs alone	53268	19.34	112995	18.47	<.0001
ICS/LABA combinations	60670	22.03	137063	22.41	<.0001
LAMA	23948	8.69	54519	8.91	<.0001
HF Medications					
ACEIs	178634	64.85	251327	41.09	<.0001
ARBs	166788	60.55	262085	42.85	<.0001
Cardioselective β-blockers	77381	28.09	124213	20.31	<.0001
Non-selective β-blockers	64739	23.50	60261	9.85	<.0001
Loop Diuretics	190593	69.20	265535	43.42	<.0001
Aldosterone antagonists	81199	29.48	77585	12.69	<.0001
Digoxins	105209	38.20	61995	10.14	<.0001
Statins	88952	32.29	186115	30.43	<.0001

Abbreviations: COPD, chronic obstructive pulmonary disease; HF, heart failure; SD, standard deviation; OPD, outpatient department; AE, acute exacerbation; SABDs, short-acting bronchodilators; LABAs, long-acting beta-agonists; LAMA, long-acting muscarinic antagonist; ICSs, inhaled corticosteroids; ACEIs, angiotensin-converting-enzyme inhibitors; ARBs, angiotensin receptor blockers.

### Risk factors for mortality in patients with COPD-heart failure overlap

**Figure 3:**
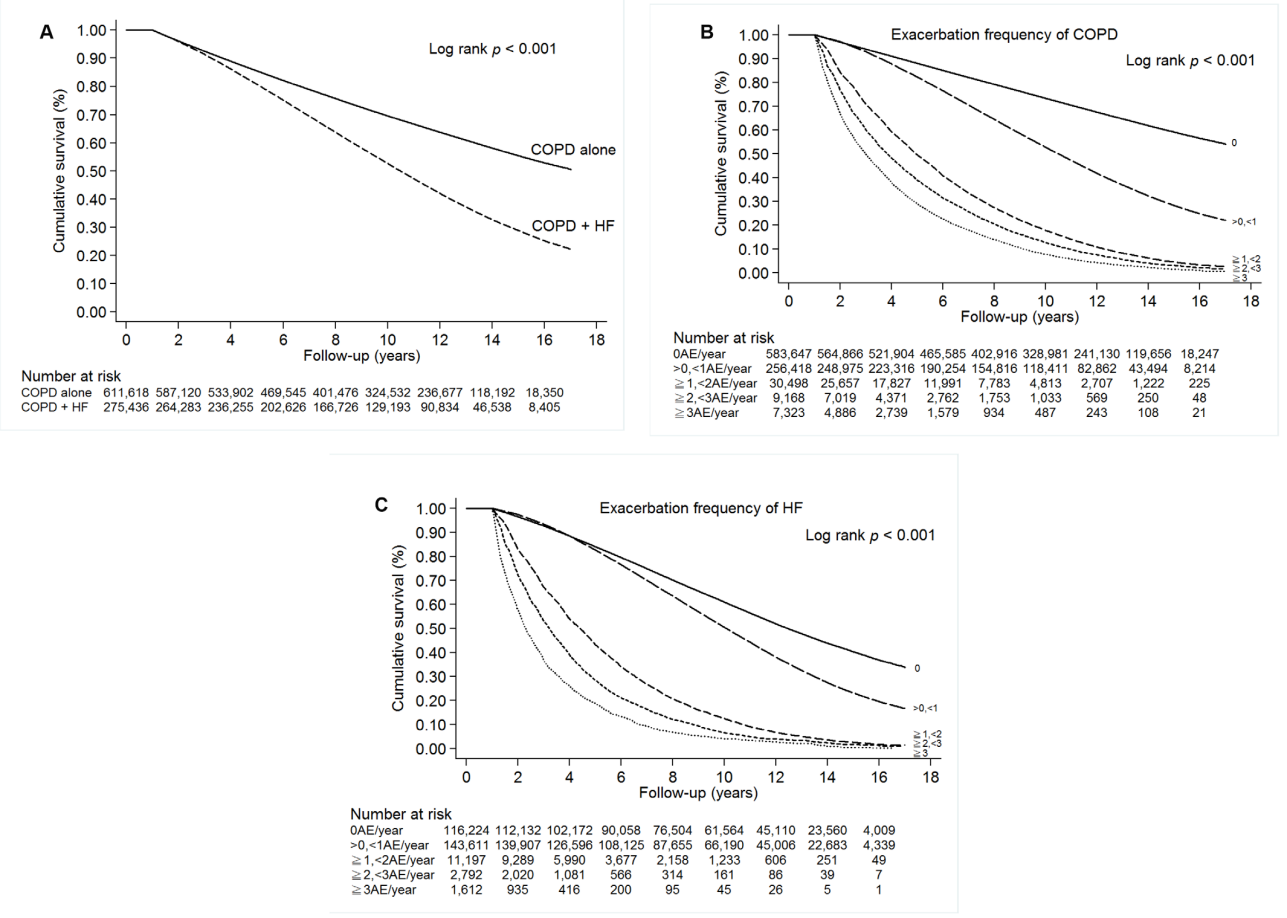
(**A**) Survival of patients with COPD alone or COPD-heart failure overlap (COPD + HF). (**B**) Survival of patients with COPD. (**C**) Survival of patients with HF.

The cumulative survival hazard was significantly lower in the COPD-heart failure overlap cohort than in the COPD alone cohort in Kaplan–Meier analysis (log-rank test, *p* < 0.001; Figure 3A). Survival decreased significantly with increasing exacerbation frequency of COPD or HF in Kaplan–Meier analysis (Figure 3B and 3C); this result was sustained after adjustment for other factors (Supplementary Table 1). We identified the following independent risk factors for mortality in the COPD-heart failure overlap cohort: age ≥ 65 years (HR, 1.59; 95% CI, 1.59–1.60), male sex (HR, 1.11; 95% CI, 1.11–1.12), diabetes mellitus (HR, 1.07; 95% CI, 1.07–1.07), hypertension (HR, 1.05; 95% CI, 1.04–1.05), cerebrovascular disease (HR, 1.06; 95% CI, 1.06–1.06), malignancy (HR, 1.24; 95% CI, 1.24–1.24), chronic kidney disease (HR, 1.03; 95% CI, 1.03–1.03), and Charlson Comorbidity Index > 1 (HR, 1.03; 95% CI, 1.03–1.03; all *p* < 0.0001; Supplementary Table 1).

### The effect of COPD medication use on mortality

In the time-dependent Cox proportional-hazards model, the use of LAMA, and ICS/LABA combinations was associated with significantly lower mortality risks (model 1: LAMA: HR = 0.76, 95% CI = 0.75–0.77; ICS/LABA combinations: HR = 0.74, 95% CI = 0.74–0.75; model 2: LAMA: HR = 0.80, 95% CI = 0.79–0.81; ICS/LABA combinations: HR = 0.77, 95% CI = 0.77–0.78; all *p <* 0.0001; Table 2). In contrast, use of ICSs alone was associated with significantly higher mortality risks. The results of subgroup analyses are presented in Figure 4A–4E.

**Table 2:** Sensitivity analyses for medication effects on mortality in COPD-heart failure overlap patients.

**Variables**	**Time-dependent model**			
	**Model 1^#^**		**Model 2^##^**	
	**HR (95% CI)**	***p***	**HR (95% CI)**	***p***
**COPD Medication**				
Non-users of SABDs	Reference		Reference	
SABDs	1.06 (1.06–1.07)	<.0001	1.06 (1.06–1.06)	<.0001
Non-users of LABAs alone	Reference		Reference	
LABAs alone	1.04 (1.03–1.04)	<.0001	1.05 (1.05–1.06)	<.0001
Non-users of ICSs alone	Reference		Reference	
ICSs alone	1.08 (1.08–1.09)	<.0001	1.09 (1.08–1.10)	<.0001
Non-users of ICS/LABA combinations	Reference		Reference	
ICS/LABA combinations	0.74 (0.74–0.75)	<.0001	0.77 (0.77–0.78)	<.0001
Non-users of LAMA	Reference		Reference	
LAMA	0.76 (0.75–0.77)	<.0001	0.80 (0.79–0.81)	<.0001
**HF Medication**				
Non-users of ACEIs	Reference		Reference	
ACEIs	1.08 (1.08–1.08)	<.0001	1.11 (1.11–1.12)	<.0001
Non-users of ARBs	Reference		Reference	
ARBs	0.76 (0.76–0.76)	<.0001	0.80 (0.79–0.80)	<.0001
Non-users of Cardioselective β-blockers	Reference		Reference	
Cardioselective β-blockers	0.72 (0.71–0.72)	<.0001	0.76 (0.76–0.77)	<.0001
Non-users of Non-selective β-blockers	Reference		Reference	
Non-selective β-blockers	0.92 (0.92–0.93)	<.0001	0.96 (0.95–0.96)	<.0001
Non-users of Loop diuretics	Reference		Reference	
Loop diuretics	1.07 (1.07–1.07)	<.0001	1.09 (1.08–1.09)	<.0001
Non-users of Aldosterone antagonists	Reference		Reference	
Aldosterone antagonists	0.96 (0.96–0.96)	<.0001	0.99 (0.98–0.99)	0.0002
Non-users of Digoxins	Reference		Reference	
Digoxins	1.13 (1.12–1.13)	<.0001	1.12 (1.12–1.13)	<.0001
Non-users of Statins	Reference		Reference	
Statins	0.75 (0.74–0.75)	<.0001	0.76 (0.76–0.77)	<.0001

Abbreviations: COPD, chronic obstructive pulmonary disease; HF, heart failure; HR, hazard ratio; CI, confidence interval; AE, acute exacerbation; SABDs, short-acting bronchodilators; LABAs, long-acting beta-agonists; LAMA, long-acting muscarinic antagonist; ICSs, inhaled corticosteroids; ACEIs, angiotensin-converting-enzyme inhibitors; ARBs, angiotensin receptor blockers.

HRs were adjusted for age, sex, income level, comorbidities, exacerbation frequency of COPD, exacerbation frequency of HF, Charlson Comorbidity Index, urbanization level and medications. All factors with *p* < 0.1 in univariate analyses were included in the Cox multivariate analysis.

^#^Medications were analyzed as time-dependent covariates (time period: 3 months).

^##^Medications were analyzed as time-dependent covariates (time period: 1 year)

**Figure 4:**
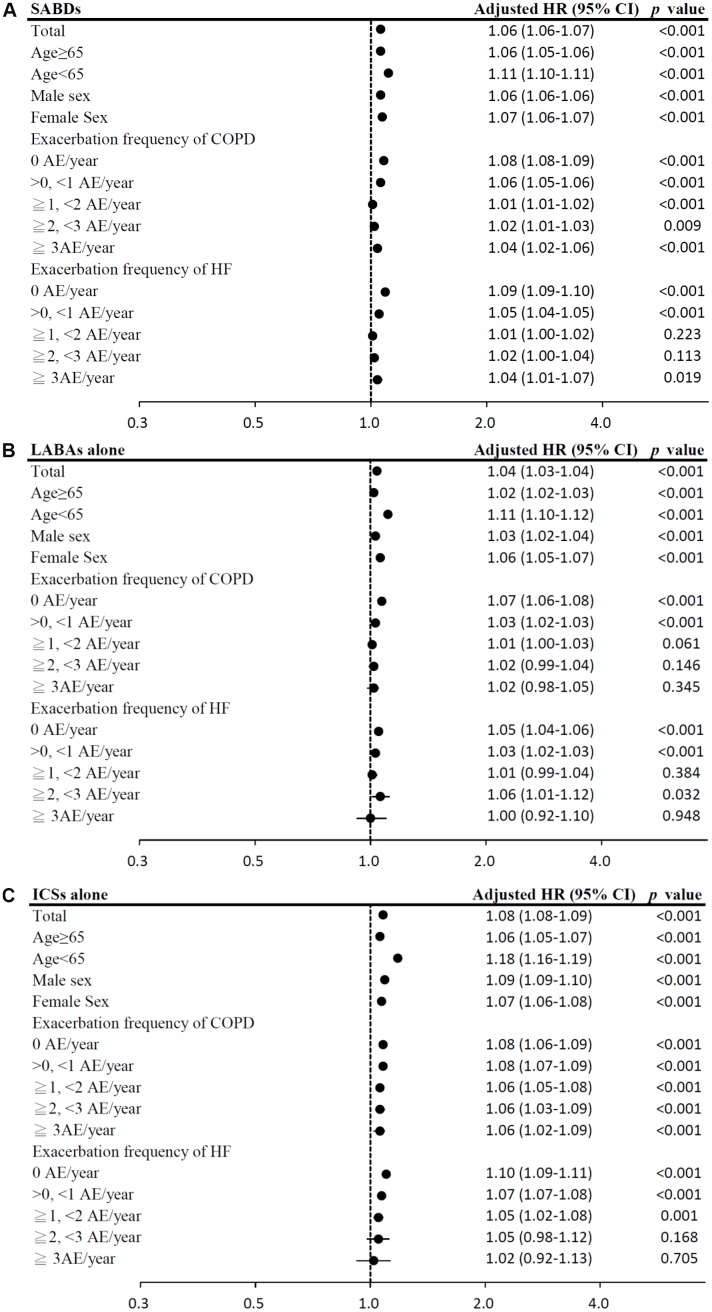
(**A**) Subgroup analysis of SABDs. (HRs were adjusted for age, sex, income level, comorbidities, exacerbation frequency of COPD, exacerbation frequency of HF, Charlson Comorbidity Index, urbanization level and medications; medications were analyzed as time-dependent covariates, time period: 3 months). (**B**) Subgroup analysis of LABAs alone. (**C**) Subgroup analysis of ICSs alone.

**Figure 4:**
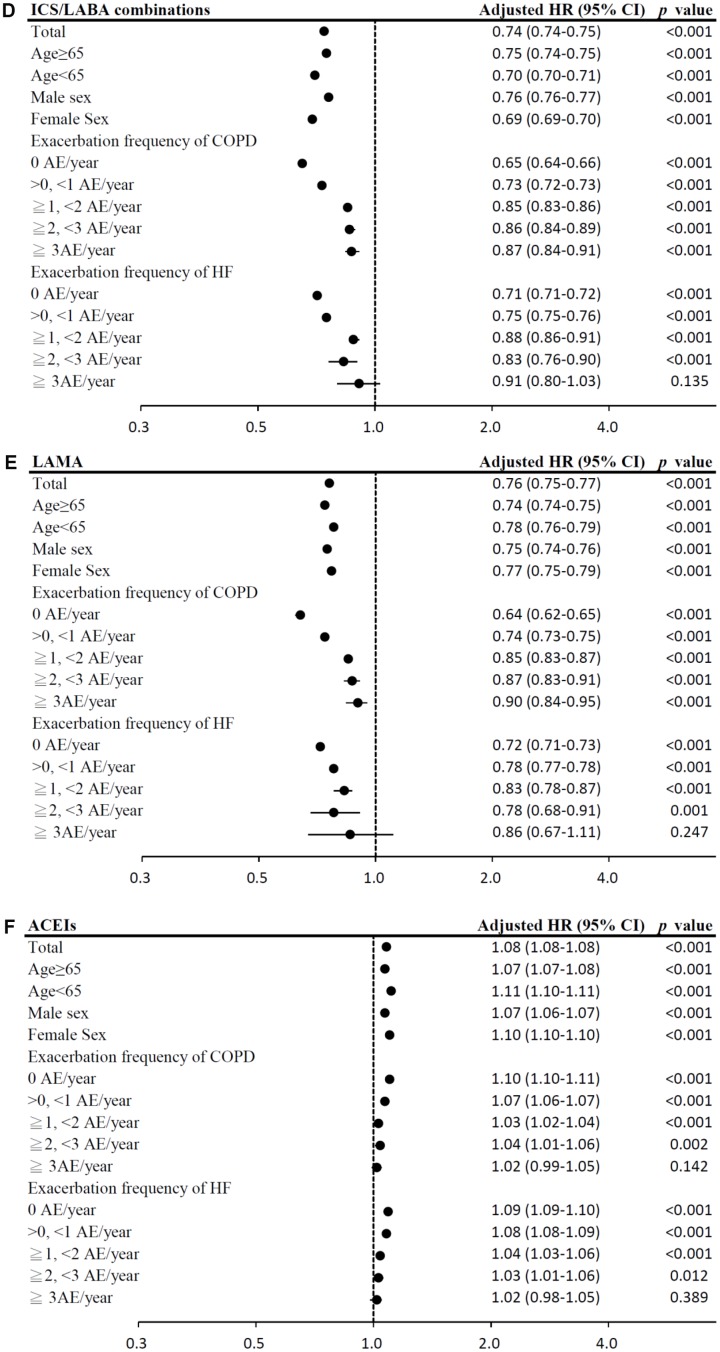
(**D**) Subgroup analysis of ICS/LABA combinations. (**E**) Subgroup analysis of LAMA. (**F**) Subgroup analysis of ACEIs.

**Figure 4:**
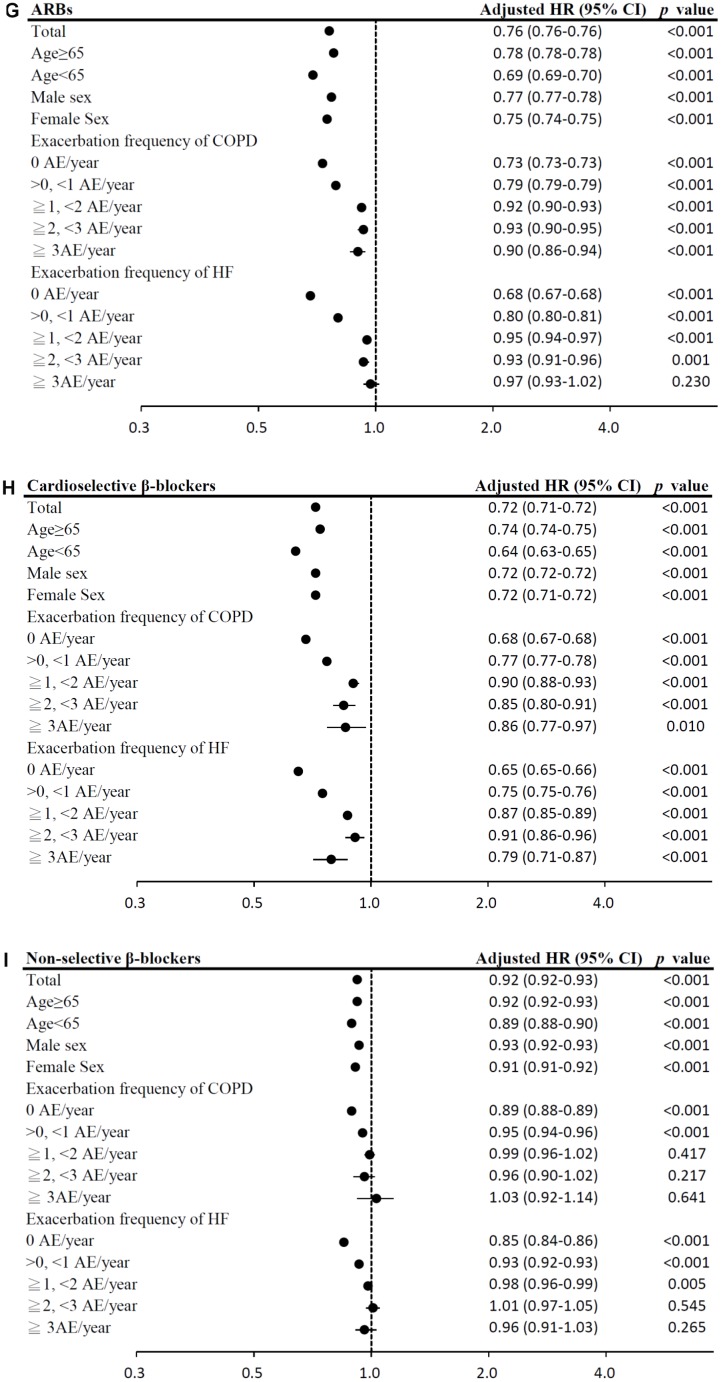
(**G**) Subgroup analysis of ARBs. (**H**) Subgroup analysis of cardioselective β blockers. (**I**) Subgroup analysis of non-selective β blockers.

**Figure 4:**
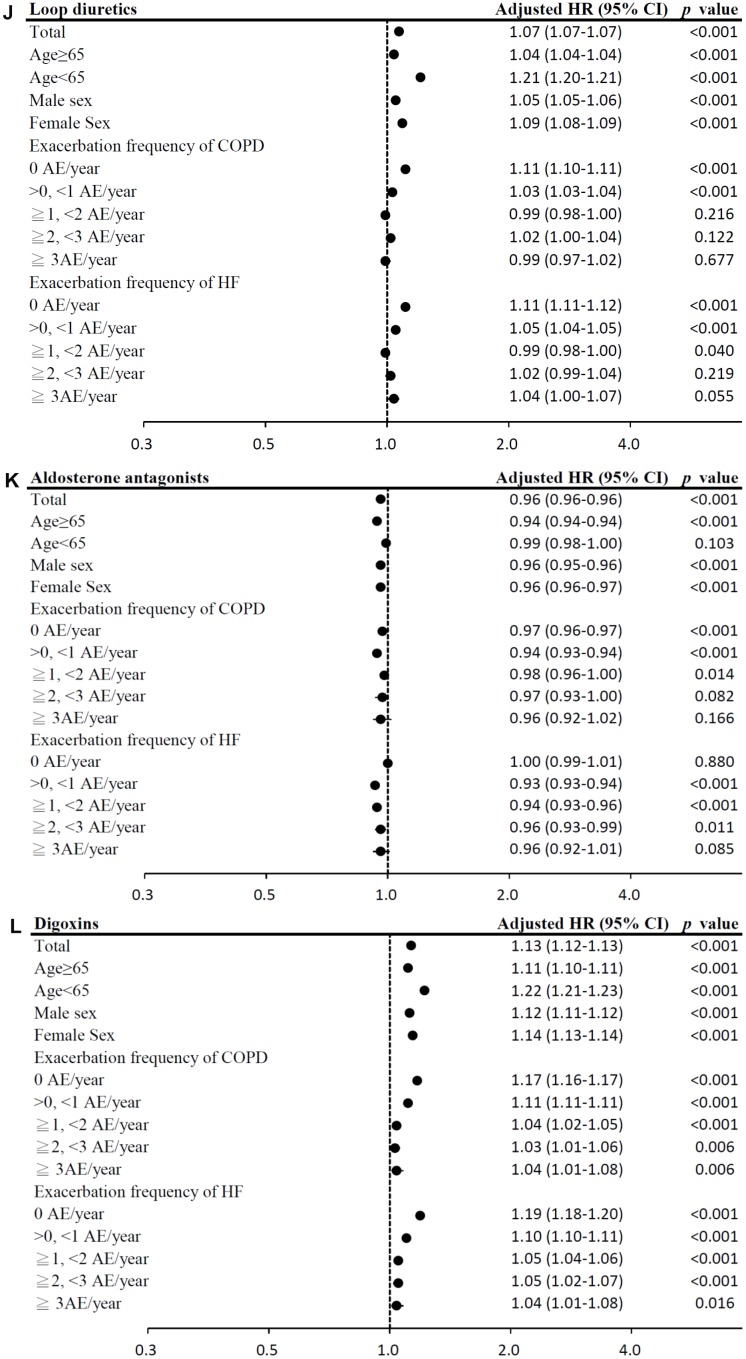
(**J**) Subgroup analysis of loop diuretics. (**K**) Subgroup analysis of aldosterone antagonists. (**L**) Subgroup analysis of digoxins.

**Figure 4:**
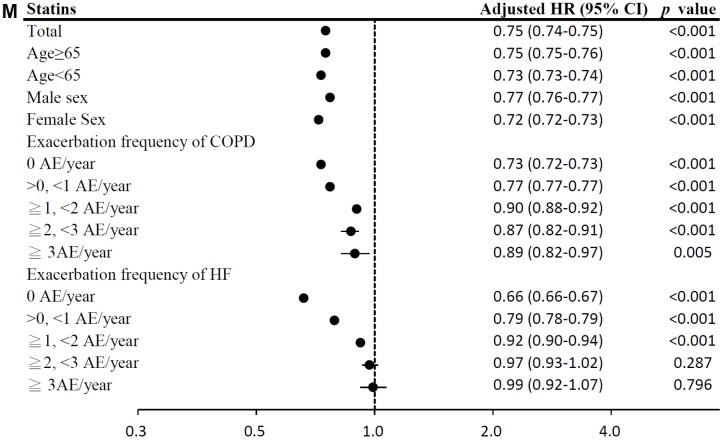
(**M**) Subgroup analysis of statin.

### The effect of HF medication use on mortality

Among HF medication, the use of ARBs, cardioselective β blockers, non-selective β blockers, aldosterone antagonists, and statins was associated with significantly lower mortality risks (model 1: ARBs: HR = 0.76, 95% CI = 0.76–0.76; cardioselective β blockers: HR = 0.72, 95% CI = 0.71–0.72; non-selective β blockers: HR = 0.92, 95% CI = 0.92–0.93; aldosterone antagonists: HR = 0.96, 95% CI = 0.96–0.96; statins: HR = 0.75, 95% CI = 0.74–0.75; model 2: ARBs: HR = 0.80, 95% CI = 0.79–0.80; cardioselective β blockers: HR = 0.76, 95% CI = 0.76–0.77; non-selective β blockers: HR = 0.96, 95% CI = 0.95–0.96; aldosterone antagonists: HR = 0.99, 95% CI = 0.98–0.99; statins: HR = 0.76, 95% CI = 0.76–0.77; all *p <* 0.001; Table 2). However, digoxins use was associated with the highest mortality risk. The results of subgroup analyses are presented in Figure 4F–4M.

### Validation of COPD and HF definitions

2,325 patients with COPD and 3,402 patients with HF were identified from the claims database of Taipei Veterans General Hospital. Then, 300 patients from each group were randomly selected for validation of diagnoses. Of them, 276 patients with COPD underwent pulmonary function test and 288 patients with HF underwent echocardiogram. Diagnoses of COPD were confirmed by pulmonologists in 282/300 subjects (94.0% sensitivity) and by pulmonary function test in 238/276 subjects (86.2% sensitivity). Diagnoses of HF were confirmed by cardiologists in 289/300 subjects (96.3% sensitivity) and by echocardiography in 263/288 subjects (91.3% sensitivity).

## DISCUSSION

This study revealed a greater medical burden in patients with COPD-heart failure overlap than in those with COPD alone. Our study demonstrated significant reductions in mortality associated with the use of LAMA, ICS/LABA combinations, ARBs, β blockers, aldosterone antagonists, and statins in patients with COPD-heart failure overlap. Results of this study, which involved large COPD-heart failure overlap and COPD cohorts – to our knowledge, the largest COPD-heart failure overlap cohort examined to date – are more valuable than those of clinical trials because they more closely reflect real-world practice. Furthermore, the definitions of COPD and HF were validated in this study, which indicated that the accuracy was excellent.

Despite the Heart Failure Society of America’s recommendation of β blocker use for HF [2], we previously documented the suboptimal use of β blockers in patients with COPD-heart failure overlap due to concern about their detrimental effects on COPD [7]. A population-based survey conducted by Lipworth *et al*. in UK enrolled 24,237 patients with HF alone and 10,853 patients with COPD-heart failure overlap, and showed that the use of β blockers in combination with ACEI/ARB was much lower in patients with COPD-heart failure overlap than in patients with HF alone (22.3% *vs.* 41.3%, *p* < 0.001) [3]. In turn, despite the Global Initiative for Chronic Obstructive Lung Disease’s recommendation of bronchodilator use in patients with COPD and COPD-heart failure overlap [1], concern about detrimental effects on HF has led to suboptimal use of these drugs [8].

This study demonstrated that LAMA, ICS/LABA combinations, ARBs, β blockers, aldosterone antagonists, and statins reduced mortality in patients with COPD-heart failure overlap. We also showed that cardioselective β blockers (bisoprolol and metoprolol) yield better survival outcomes than do non-selective β blockers in patients with COPD-heart failure overlap. Pulmonary effects appear to differ among β blockers; non-selective, but not cardioselective, β blockers have been found to reduce forced expiratory volume in 1 second (FEV_1_) and to increase airway hypersensitiveness in patients with COPD [9]. In a randomized cross-over study [10], bisoprolol is better tolerated than carvedilol on lung function in patients with moderate to severe COPD.

Paradoxically, as evidence for the efficacy of β blockers in COPD accumulates, studies have shown adverse cardiovascular effects of β agonists. The cardiac safety of LABA treatment, reported in clinical trials, is a result of administration by inhalation, which limits the transition of active drugs into the systemic circulation. However, inhaled β agonists still reach the systemic circulation through pulmonary circulation or absorption *via* the gastrointestinal tract after the swallowing of residual particles [11]. β agonists are associated with increased mortality and HF-related hospitalization in patients with existing HF [12]. A meta-analysis of randomized placebo-controlled trials showed that β-agonist treatment significantly increased the risk of a cardiovascular event in patients with obstructive airway disease compared with placebo (relative risk [RR] = 2.54; 95% CI, 1.59–4.05) [13]. Not surprisingly, SABDs and LABAs alone use was associated with increased risk of mortality in this study. Tiotropium, an inhaled LAMA approved for COPD maintenance treatment that was examined in this study, was associated with lower mortality than placebo in clinical trial [14]. In clinical trial [15], use of ICS/LABA combination over three years showed a decrease in mortality that almost reached statistical significance (HR=0.825, *p* = 0.052). A meta-analysis of randomized controlled trials showed that ICS/LABA combinations use was associated with a lower risk of death compared with use of ICSs alone [16]. According to international guideline for COPD [1], ICS monotherapy is not recommended for the management of COPD. Among older patients with COPD, ICS/LABA combinations use was associated with a lower risk of death compared with use of LABAs alone [17]. Sensitivity and subgroup analyses demonstrated that the benefits of LAMA and ICS/LABA combinations in patients with COPD and HF were consistent.

The most common side effect of ACEI therapy is cough, the prevalence of which approaches 50% in Chinese populations [18]. The incidence of ACEI-related cough has been reported to be 2.7 folds higher in Chinese subjects compared with Caucasians [19]. ACEI-induced cough may represent bronchial hyperresponsiveness in some patients [5]. ARBs do not appear to induce cough or bronchial hyperreactivity in patients with symptomatic asthma [20]. Indeed, bronchial hyperresponsiveness is an independent predictor of mortality in COPD patients [6]. In this study, almost all enrollees were Taiwanese. Our study showed that ARBs use is associated with better survival outcomes than ACEIs use. Similarly, use of statins (RR = 0.53; 95% CI, 0.43–0.65) and ARBs (RR = 0.62; 95% CI, 0.44–0.87), but not ACEIs (RR = 1.04; 95% CI, 0.86–1.25), was associated with survival benefit in 22,784 patients with COPD and high cardiovascular risk in a time-matched nested case-control study [21]. In a double-blind, placebo-controlled, randomized controlled trial [22], 80 patients with at least moderate COPD were randomized to either 10 weeks of therapy with an ACEI (10 mg enalapril) or placebo. Use of the ACEI significantly reduced the peak power response to exercise training in COPD patients. Recently, a population-based cohort study [23] also showed that statins use was associated with a lower mortality risk in patients with COPD.

In this study, use of SABDs, loop diuretics, and digoxins increased mortality risk in patients with COPD-heart failure overlap. Possible explanations for the higher mortality risk associated with SABD use are related to various cardiac side effects. In addition, excess SABD, loop diuretic, and digoxin use has been associated with poor control of COPD and HF, which may itself be the cause of this increased risk. Regardless of whether the association demonstrated is causal, however, chronic use of these drugs is a very powerful marker of high mortality risk in patients with COPD-heart failure overlap. Such patients warrant special attention.

This study was strengthened by using a nationwide population-based data to conduct a real-world study, which enrolled nearly all cases of COPD and HF in Taiwan with minimal selection and referral biases, as the NHI coverage rate is >99% and all cardiopulmonary-related healthcare is covered. To minimize immortal time bias, medication use was analyzed using time-dependent covariates in the Cox proportional-hazards model. Nevertheless, this study has some limitations. First, the claim-based dataset omitted some personal information, such as data on smoking and obesity. Although these factors appear to have no influence on medication selection and we made every effort to correct for confounding factors. Second, we could not assess drug adherence directly. However, this bias is toward the null hypothesis and would lead to underestimation of the actual effects of medications. Finally, the external validity of the results of this study should be of concern because all enrollees were Taiwanese. The generalizability of our findings to non-Asian populations must be further verified.

## METHODS

### Data Source

This cohort study used the Taiwan National Health Insurance Research Database (NHIRD), which is derived from the claims data of the Taiwan’s National Health Insurance (NHI) program, initiated in 1995, from more than 99% of Taiwan’s 23 million residents [24]. The NHIRD, provided for research purposes by Taiwan’s National Health Research Institute, contains healthcare data from NHI participants, including information on demographic characteristics; medical diagnoses, procedure, expenditure, and all detailed prescriptions. The accuracy of diagnoses recorded in the NHIRD, such as sleep apnea [24], pneumonia [24], asthma [25], COPD [25], tuberculosis contact [26], and tuberculosis [27] has been validated in our previous work. The study was exempted from full review by the Institutional Review Board of Taipei City Hospital because the datasets consisted of de-identified secondary data.

### Study design and population

In this nationwide cohort study, we retrospectively analyzed medication effects on mortality in patients with COPD-heart failure overlap. All patients with COPD diagnoses recorded in the NHIRD between 1 January 1997 and 31 December 2012 were divided into the COPD alone and COPD-heart failure overlap cohorts. COPD and HF were identified using diagnostic codes from the International Classification of Diseases, 9^th^ Revision, Clinical Modification (HF: 425.4, 425.9, 402.01, 402.11, 402.91, 404.01, 404.03, 404.11, 404.13, 404.91, 404.93, and 428.xx; COPD: 491.xx, 492.xx, and 496.xx). Patients with at least six recorded outpatient visits or one hospitalization for either disease were enrolled and followed until the date of death, withdrawal from insurance, or 31 December 2013. Information on the use of medications recommended in guidelines for patients with COPD or HF were extracted [1, 28]. Patients with follow-up periods < 1 year, those aged < 40 years, and those who were not prescribed medications for COPD or HF were excluded.

### Potential confounders and severity classification

In the investigation of the effects of medications on mortality, age, sex, urbanization level, income level, Charlson Comorbidity Index, exacerbation frequency of COPD, exacerbation frequency of HF, and comorbidities were considered to be potential confounders. Because the most important factor in classifying COPD severity appears to be a previous history of exacerbations [29], exacerbation frequency of COPD was determined using the annual frequency of hospitalizations and emergency department visits for acute exacerbation during the follow-up period. Exacerbation frequency of HF was classified in the same way and the negative correlation between COPD/HF severity and cumulative survival has been validated in our previous study [7]. Based on the number of acute exacerbations (AE) per year, patients were assigned to the 0 AE/year, >0 and <1 AE/year, ≥1 and <2 AE/year, ≥2 and <3 AE/year, and ≥3 AE/year groups.

### Exposure to COPD and HF medications

Patients who took ≥28 days of each medication recommended in guidelines for patients with COPD or HF during the follow-up period, were assigned to respective medication groups. Prescriptions of medications for COPD or HF were assessed during each time period (3 months or 1 year) for each individual patient [30]. A 3-month period was chosen, because medications are usually prescribed for 3 months at a time. A 1-year period was used to recheck the robustness of the results of 3-month period. COPD medication groups were: short-acting bronchodilators (SABDs), including short-acting β2 agonists (albuterol, fenoterol, terbutaline, procaterol) and short-acting muscarinic antagonists (ipratropium); long-acting β2 agonists (LABAs; salmeterol, formoterol, bambuterol, indacaterol); long-acting muscarinic antagonist (LAMA; tiotropium); ICSs (budesonide, fluticasone, beclomethasone) and ICS/LABA combinations (fluticasone/salmeterol, budesonide/formoterol, beclomethasone/formoterol). HF medication groups were: ACEIs (benazepril, captopril, enalapril, fosinopril, lisinopril, perindopril, perindopril, quinapril, ramipril), ARBs (losartan, candesartan, valsartan, irbesartan, telmisartan, eprosartan, olmesartan, azilsartan), cardioselective β blockers (bisoprolol, metoprolol), non-selective β blockers (carvedilol), loop diuretics (furosemide, bumetanide), aldosterone antagonists (spironolactone, eplerenone); digoxins (digoxin, digitoxin), and statins (atorvastatin, fluvastatin, lovastatin, pitavastatin, pravastatin, rosuvastatin, simvastatin).

### Validation

The claims database of Taipei Veterans General Hospital (a 3,035-bed tertiary referral hospital in Taiwan) was used to validate the accuracy of the diagnoses of COPD and HF in this study. The contents of this database are used for reimbursement and are similar to those of the NHIRD [24–27]. The diagnoses of COPD and HF were validated by analysis of selected samples from this database using the same criteria. COPD was defined as COPD diagnosed by a pulmonologist, or fixed airflow limitation determined by pulmonary function test [1]. HF was defined as HF diagnosed by a cardiologist, or inadequate cardiac output determined by echocardiography [2]. Random sampling of claims data for validation was performed using SPSS version 20.

### Statistical analyses

Data entry and analysis were performed using SAS statistical software (version 9.4; SAS Institute, Cary, NC, USA). Descriptive analysis was used to compare the COPD alone group and COPD-heart failure overlap group in terms of demographic data and the prescription medications during the study period. All the data were presented as mean ± standard deviation (SD) or percentage (%). Comparison between two groups was made by independent Student’s t-test for continuous variables or Pearson’s chi-square test for categorical variables as appropriate. Kaplan-Meier survival analysis and the log-rank test were used to examine the difference between the cohorts. We used the Cox proportional-hazards regression models to determine the survival effect of medication use during the follow-up period. Medication prescriptions in each 3-month and 1-year period served as time-dependent covariates. Patients who were prescribed ≥28 days of each medication within each interval were assigned to the respective medication groups and all others were defined as non-users [25]. Other baseline variables were analyzed as non–time-dependent covariates. All factors with *p* values < 0.1 in univariate analyses were included in the Cox multivariate analysis. The hazard ratio (HR) and 95% confidence intervals (CIs) were calculated after adjustment for possible confounding factors. Two-tailed *p* values < 0.05 were considered statistically significant. Subgroup analyses were performed according to age, sex, exacerbation frequency of COPD and HF.

## CONCLUSIONS

This study demonstrated that patients with COPD-heart failure overlap have more outpatient visits per year, more hospitalization, and a shorter time to mortality than do patients with COPD alone. LAMA, ICS/LABA combination, ARB, β blocker, aldosterone antagonist, and statin use decreased the mortality risk.

## Supplementary Material

Supplementary Table 1
