# Correction for: Real-world effectiveness of medications on survival in patients with COPD-heart failure overlap

**DOI:** 10.18632/aging.102363

**Published:** 2019-10-07

**Authors:** Vincent Yi-Fong Su, Yao-Hsu Yang, Diahn-Warng Perng, Ying-Huang Tsai, Kun-Ta Chou, Kang-Cheng Su, Wei-Juin Su, Pau-Chung Chen, Kuang-Yao Yang

**Affiliations:** 1 Department of Internal Medicine, Taipei City Hospital, Taipei City Government, Taipei, Taiwan; 2 Department of Chest Medicine, Taipei Veterans General Hospital, Taipei, Taiwan; 3 Faculty of Medicine, School of Medicine, National Yang-Ming University, Taipei, Taiwan; 4 Institute of Clinical Medicine, National Yang-Ming University, Taipei, Taiwan; 5 Institute of Emergency and Critical Care Medicine, National Yang-Ming University, Taipei, Taiwan; 6 Cancer Progression Research Center, National Yang-Ming University, Taipei, Taiwan; 7 Division of Pulmonary and Critical Care Medicine and Department of Respiratory Care, Chang Gung Memorial Hospital, Chiayi, Taiwan; 8 Health Information and Epidemiology Laboratory, Chang Gung Memorial Hospital, Chiayi, Taiwan; 9 Department of Traditional Chinese Medicine, Chang Gung Memorial Hospital, Chiayi, Taiwan; 10 School of Traditional Chinese Medicine, College of Medicine, Chang Gung University, Taoyuan, Taiwan; 11 Department of Respiratory Therapy, Chang Gung University, Taoyuan, Taiwan; 12 Institute of Occupational Medicine and Industrial Hygiene, National Taiwan University College of Public Health, Taipei, Taiwan; 13 Department of Environmental and Occupational Medicine, National Taiwan University Hospital and National Taiwan University College of Medicine, Taipei, Taiwan

**Keywords:** correction

**This article has been corrected:** Due to production mistake the wrong Figure 2 was posted in this paper. The authors declare that this correction does not change the results or conclusions of this paper. The correct Figure 2 is provided below.

Original article: Aging. 2019; 11:3650–3667. 3650-3667 . https://doi.org/10.18632/aging.102004

**Figure 2:**
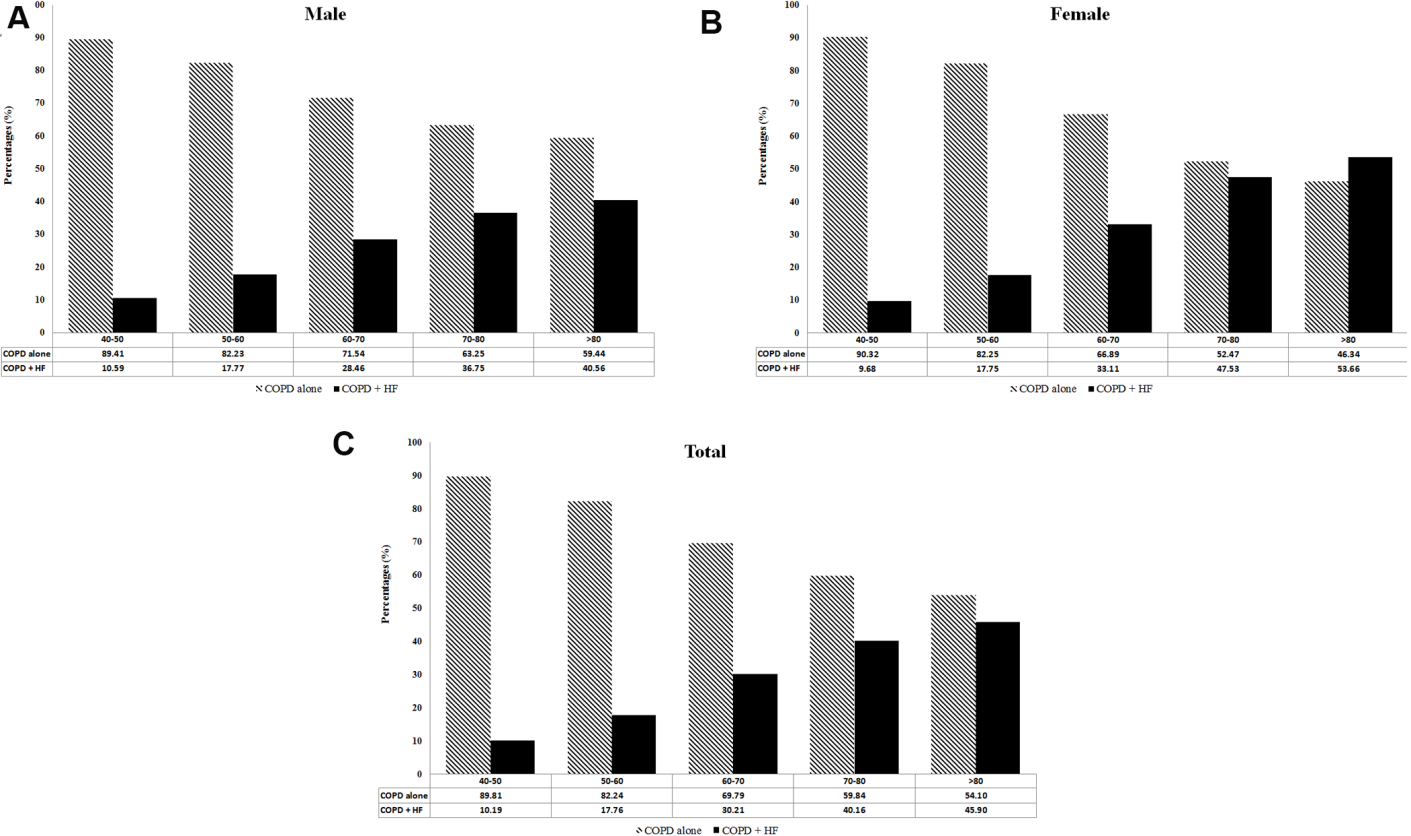
(**A**) Percentage of patients with COPD-heart failure overlap (COPD+HF) in different age groups. (**B**) Percentage of patients with COPD-heart failure overlap (COPD+HF) in different age groups in male sex. (**C**) Percentage of patients with COPD-heart failure overlap (COPD+HF) in different age groups in female sex.

