# Supplementary material for: Real-world effectiveness of medications on survival in patients with COPD-heart failure overlap
Source: Aging (Albany NY). 2019 Jun 7;11(11):3650–67. doi: 10.18632/aging.102004 (PMC6594806; doi:10.18632/aging.102004)
Supplement: Supplementary Table 1 [file aging-11-102004-s001.docx]

SUPPLEMENTARY TABLE

| Supplementary Table 1. Analyses of risk factors for mortality in COPD-heart failure overlap patients by Cox proportional hazards regression analysis. | | | | | | | |
| --- | --- | --- | --- | --- | --- | --- | --- |
| **Variables** |  | | **Unadjusted** | |  | **Adjusted*** | |
|  |  |  | **HR (95% CI)** | ***p*** |  | **HR (95% CI)** | ***p*** |
| Age ≥ 65 years |  | 1.90 (1.90-1.91) | | <.0001 |  | 1.59 (1.59-1.60) | <.0001 |
| Male gender |  | 1.17 (1.17-1.17) | | <.0001 |  | 1.11 (1.11-1.12) | <.0001 |
| Income level |  |  | |  |  |  |  |
| 0 |  | Referent | |  |  | Referent |  |
| 1–15840 |  | 1.00 (1.00-1.00) | | 0.1208 |  | 0.92 (0.91-0.92) | <.0001 |
| 15841–25000 |  | 0.86 (0.85-0.86) | | <.0001 |  | 0.90 (0.90-0.90) | <.0001 |
| ≧25000 |  | 0.55 (0.55-0.56) | | <.0001 |  | 0.74 (0.74-0.74) | <.0001 |
| Comorbidities |  |  | |  |  |  |  |
| Diabetes mellitus |  | 1.04 (1.04-1.04) | | <.0001 |  | 1.07 (1.07-1.07) | <.0001 |
| Hypertension |  | 1.07 (1.07-1.07) | | <.0001 |  | 1.05 (1.04-1.05) | <.0001 |
| Cerebrovascular disease |  | 1.12 (1.12-1.12) | | <.0001 |  | 1.06 (1.06-1.06) | <.0001 |
| Ischemia heart disease |  | 0.99 (0.99-0.99) | | <.0001 |  | 0.96 (0.95-0.96) | <.0001 |
| Malignancy |  | 1.21 (1.20-1.21) | | <.0001 |  | 1.24 (1.24-1.24) | <.0001 |
| Cirrhosis |  | 0.85 (0.85-0.85) | | <.0001 |  | 0.90 (0.90-0.90) | <.0001 |
| Chronic kidney disease |  | 1.23 (1.23-1.24) | | <.0001 |  | 1.03 (1.03-1.03) | <.0001 |
| Exacerbation frequency of COPD (AE/year) |  |  | |  |  |  |  |
| 0 AE/year |  | Ref | |  |  | Ref |  |
| >0, <1 AE/year |  | 1.47 (1.47-1.47) | | <.0001 |  | 1.26 (1.26-1.27) | <.0001 |
| ≧1, <2 AE/year |  | 1.95 (1.94-1.95) | | <.0001 |  | 1.52 (1.51-1.53) | <.0001 |
| ≧2, <3 AE/year |  | 1.99 (1.97-2.00) | | <.0001 |  | 1.51 (1.50-1.52) | <.0001 |
| ≧ 3AE/year |  | 1.99 (1.98-2.01) | | <.0001 |  | 1.51 (1.49-1.52) | <.0001 |
| Exacerbation frequency of HF (AE/year) |  |  | |  |  |  |  |
| 0 AE/year |  | Ref | |  |  | Ref |  |
| >0, <1 AE/year |  | 1.48 (1.47-1.48) | | <.0001 |  | 1.31 (1.31-1.32) | <.0001 |
| ≧1, <2 AE/year |  | 1.99 (1.98-2.00) | | <.0001 |  | 1.54 (1.54-1.55) | <.0001 |
| ≧2, <3 AE/year |  | 1.99 (1.97-2.01) | | <.0001 |  | 1.47 (1.45-1.48) | <.0001 |
| ≧ 3AE/year |  | 1.97 (1.94-1.99) | | <.0001 |  | 1.44 (1.42-1.46) | <.0001 |
| Charlson Comorbidity Index |  |  | |  |  |  |  |
| 0–1 |  | Referent | |  |  | Referent |  |
| >1 |  | 1.07 (1.07-1.08) | | <.0001 |  | 1.03 (1.03-1.03) | <.0001 |
| Urbanization |  |  | |  |  |  |  |
| I (Highest) |  | Referent | |  |  | Referent |  |
| II |  | 1.02 (1.02-1.02) | | <.0001 |  | 1.01 (1.01-1.01) | <.0001 |
| III |  | 1.08 (1.08-1.08) | | <.0001 |  | 1.05 (1.04-1.05) | <.0001 |
| IV (Lowest) |  | 1.08 (1.08-1.09) | | <.0001 |  | 1.07 (1.06-1.07) | <.0001 |
| COPD Medications^&^ |  |  | |  |  |  |  |
| SABDs |  | 1.13 (1.13-1.13) | | <.0001 |  | 1.06 (1.06-1.07) | <.0001 |
| LABAs alone |  | 1.14 (1.13-1.15) | | <.0001 |  | 1.04 (1.03-1.04) | <.0001 |
| ICSs alone |  | 1.22 (1.21-1.22) | | <.0001 |  | 1.08 (1.08-1.09) | <.0001 |
| ICS/LABA combinations |  | 0.69 (0.69-0.69) | | <.0001 |  | 0.74 (0.74-0.75) | <.0001 |
| LAMAs |  | 0.69 (0.69-0.70) | | <.0001 |  | 0.76 (0.75-0.77) | <.0001 |
| HF Medications^&^ |  |  | |  |  |  |  |
| ACEIs |  | 1.18 (1.18-1.18) | | <.0001 |  | 1.08 (1.08-1.08) | <.0001 |
| ARBs |  | 0.66 (0.65-0.66) | | <.0001 |  | 0.76 (0.76-0.76) | <.0001 |
| Cardioselective β-blockers |  | 0.57 (0.57-0.57) | | <.0001 |  | 0.72 (0.71-0.72) | <.0001 |
| Non-selective β-blockers |  | 0.83 (0.83-0.84) | | <.0001 |  | 0.92 (0.92-0.93) | <.0001 |
| Loop diuretics |  | 1.08 (1.08-1.09) | | <.0001 |  | 1.07 (1.07-1.07) | <.0001 |
| Aldosterone antagonists |  | 0.98 (0.97-0.98) | | <.0001 |  | 0.96 (0.96-0.96) | <.0001 |
| Digoxins |  | 1.21 (1.21-1.21) | | <.0001 |  | 1.13 (1.12-1.13) | <.0001 |
| Statins |  | 0.59 (0.59-0.59) | | <.0001 |  | 0.75 (0.74-0.75) | <.0001 |
| Abbreviations: COPD, chronic obstructive pulmonary disease; HF, heart failure; HR, hazard ratio; CI, confidence interval; AE, acute exacerbation; SABDs, short-acting bronchodilators; LABAs, long-acting beta-agonists; LAMA, long-acting muscarinic antagonist; ICSs, inhaled corticosteroids; ACEIs, angiotensin-converting-enzyme inhibitors; ARBs, angiotensin receptor blockers.  HRs were adjusted for age, sex, income level, comorbidities, exacerbation frequency of COPD, exacerbation frequency of HF, Charlson Comorbidity Index, urbanization level and medications.  *All factors with p < 0.1 in univariate analyses were included in the Cox multivariate analysis.  ^&^Medications were analyzed as time-dependent covariates (time period: 90 days). | | | | | | | |
